# Opportunities for immunotherapy in microsatellite instable colorectal cancer

**DOI:** 10.1007/s00262-016-1832-7

**Published:** 2016-04-08

**Authors:** Harm Westdorp, Felix L. Fennemann, Robbert D. A. Weren, Tanya M. Bisseling, Marjolijn J. L. Ligtenberg, Carl G. Figdor, Gerty Schreibelt, Nicoline Hoogerbrugge, Florian Wimmers, I. Jolanda M. de Vries

**Affiliations:** 1 Department of Tumor Immunology, Radboud Institute for Molecular Life Sciences, Radboud University Medical Center, Nijmegen, The Netherlands; 2 Department of Medical Oncology, Radboud University Medical Center, Geert Grooteplein 26, 6525 GA Nijmegen, The Netherlands; 3 Department of Human Genetics, Radboud University Medical Center, Nijmegen, The Netherlands; 4 Department of Gastroenterology, Radboud University Medical Center, Nijmegen, The Netherlands; 5 Department of Pathology, Radboud University Medical Center, Nijmegen, The Netherlands

**Keywords:** Microsatellite instability, Frameshift-derived neoantigens, Colorectal cancer, Lynch syndrome, Immunotherapy, CIMT 2015

## Abstract

Microsatellite instability (MSI), the somatic accumulation of length variations in repetitive DNA sequences called microsatellites, is frequently observed in both hereditary and sporadic colorectal cancer (CRC). It has been established that defects in the DNA mismatch repair (MMR) pathway underlie the development of MSI in CRC. After the inactivation of the DNA MMR pathway, misincorporations, insertions and deletions introduced by DNA polymerase slippage are not properly recognized and corrected. Specific genomic regions, including microsatellites, are more prone for DNA polymerase slippage and, therefore, more susceptible for the introduction of these mutations if the DNA MMR capacity is lost. Some of these susceptible genomic regions are located within the coding regions of genes. Insertions and deletions in these regions may alter their reading frame, potentially resulting in the transcription and translation of frameshift peptides with c-terminally altered amino acid sequences. These frameshift peptides are called neoantigens and are highly immunogenic, which explains the enhanced immunogenicity of MSI CRC. Neoantigens contribute to increased infiltration of tumor tissue with activated neoantigen-specific cytotoxic T lymphocytes, a hallmark of MSI tumors. Currently, neoantigen-based vaccination is being studied in a clinical trial for Lynch syndrome and in a trial for sporadic MSI CRC of advanced stage. In this Focussed Research Review, we summarize current knowledge on molecular mechanisms and address immunological features of tumors with MSI. Finally, we describe their implications for immunotherapeutic approaches and provide an outlook on next-generation immunotherapy involving neoantigens and combinatorial therapies in the setting of MSI CRC.

## Colorectal cancer

Colorectal cancer (CRC) is still the third leading cause of cancer-related death worldwide [1]. However, the incidence of CRC decreased at least 2 % per year between 1998 and 2010 [2]. The decreased incidence of CRC is strongly related to the identification of novel risk factors and improved clinical management. For example, screenings by colonoscopy contributed to an estimated overall decline in CRC incidence and mortality of 11 and 14 %, respectively, within a time period of 25 years [3]. Despite these improvements, it has been estimated that approximately 1,360,000 new CRC cases will be diagnosed worldwide, and the number of CRC-related deaths has been projected to be almost 700,000 [1].

CRC is divided into sporadic, familial or hereditary cases based on the etiology of the disease. In sporadic CRC cases, accounting for approximately 70–75 % of all CRC cases, environmental lifestyle risk factors, including obesity, smoking and alcohol consumption, may have contributed to the development of the CRC, but no heritable germline aberrations are expected to be involved [4]. In sharp contrast, heritable predisposing germline aberrations are expected or known to be involved in familial and hereditary CRC, respectively. Familial CRC, contributing to approximately 20 % of all the CRC cases, is characterized by a positive family history and an increased familial risk of CRC, but the causative, possibly multiple less penetrant, germline aberrations are undefined [5]. In contrast, in 5–10 % of all CRC cases referred to as hereditary CRC, a high-penetrant CRC predisposing germline aberration is observed. To date, several high-penetrant CRC predisposing syndromes have been identified and due to the heritability of the causative germline aberration, entire families are at an increased lifetime risk of CRC [5, 6].

## High-penetrant CRC predisposing syndromes

Hereditary CRC predisposing syndromes can be divided in two subgroups based on the presence or absence of polyposis, namely polyposis and CRC predisposing syndromes and non-polyposis CRC predisposing syndromes. Polyposis is defined as the presence of tens to thousands of premalignant polyps in the colorectum. The majority of polyposis and CRC predisposing syndromes are inherited in an autosomal dominant manner, including familial adenomatous polyposis [7, 8] and polymerase proofreading-associated polyposis [9]. Familial adenomatous polyposis is caused by germline mutations in *APC* [7, 8], and polymerase proofreading-associated polyposis is predisposed by germline mutations in the proof reading domain of *POLE* and *POLD1* [9]. To date, two autosomal recessive polyposis syndromes have been identified, *MUTYH*-associated polyposis [10] and *NTHL1*-associated polyposis [11]. In both *MUTYH*- as well as *NTHL1*-associated polyposis, the base excision repair pathway is affected. This hampers the recognition and correction of damaged bases and, consequently, results in the accumulation of base substitutions. In contrast to the previous syndromes, the development of polyps is rarely observed in patients diagnosed with Lynch syndrome (LS), previously known as hereditary non-polyposis colorectal cancer [12–14]. LS is now recognized as the most prevalent hereditary CRC predisposing syndrome, explaining approximately 2–7 % of the CRC cases diagnosed [15].

## Lynch syndrome

LS is an autosomal dominantly inherited syndrome caused by monoallelic germline aberrations affecting one of the DNA mismatch repair (MMR) genes. Germline mutations in the *MSH2* locus were the first identified genomic aberrations predisposing to LS [16]; thereafter, the predisposing role of germline aberrations in the DNA MMR genes *MLH1 *[17], *MSH6 *[18], and *PMS2 *[19] was established. In addition, a novel LS predisposing molecular mechanism has been identified. It has been demonstrated that germline deletions affecting the 3′ exon of *EPCAM* result in transcriptional read-through and induce epigenetic silencing of the downstream *MSH2* locus by promoter hypermethylation [20].

The lifetime risk of CRC in LS patients is strongly associated with the causative gene/germline defect. The cumulative risk of CRC by the age of 70 years is higher in Lynch patients with pathogenic germline mutations in *MSH2* (48–77 %), *MLH1* (41–79 %) or deletions affecting the 3′ exon of *EPCAM* (69–75 %), compared to carriers of pathogenic germline mutations in *MSH6* (12-50 %) or *PMS2* (15–20 %) [21–23]. In addition, LS patients are at an increased risk to develop extracolonic malignancies in the endometrium, ovaries, stomach, small intestines, urinary tract and sebaceous glands [6]. Similar to the risk of CRC, the cumulative risk to develop these extracolonic malignancies is associated with the causative gene/germline defect (reviewed in [24]).

The role of the DNA MMR pathway on the development of CRC in LS patients has been well established. In MMR-proficient cells, the DNA MMR proteins MLH1, MSH2, MSH6 and PMS2 can form different heterodimeric protein complexes. MMR proteins recognize and correct misincorporations, insertions and deletions introduced by DNA polymerase slippage. These replication errors are strongly associated with the low fidelity of DNA polymerases, especially in repetitive DNA sequences like microsatellites [25]. If, according to Knudson’s second-hit model [26], the remaining wild-type allele is somatically inactivated in LS patients, the DNA MMR capacity is lost. Mutations arise since these replication errors are not properly recognized and corrected anymore. This will lead to the development of CRC with microsatellite instability (MSI) [27]. This strong correlation between the development of MSI CRC and LS has been well established: virtually all CRC derived from LS patients have MSI. In addition, especially in CRC patients below the age of 50 years, MSI is used as a biomarker for the identification of LS patients [6, 28, 29].

## Sporadic MSI CRC

Next to CRC derived from LS patients, MSI is encountered in approximately 15–20 % of the CRC derived from sporadic CRC patients [30, 31]. Therefore, the vast majority of all the MSI CRC are considered sporadic since only 2–3 % of all CRC come from LS patients with germline mutations in one of the DNA MMR genes [32]. Similar to CRC derived from LS patients [33, 34], these tumors have MSI and are mostly chromosomal stable [35].

Sporadic MSI CRC share histological features as mucinous differentiation and stromal inflammatory reactions with CRC derived from LS patients [36]. Similar to the improved prognosis for LS patients compared to sporadic CRC [37], a better prognosis is reported if sporadic CRC have MSI [38].

Since no germline aberrations affecting one of the DNA MMR genes are present in patients with sporadic MSI CRC, the MMR pathway is inactivated in a different manner compared to the previously discussed mechanism in LS patients. The most frequently observed molecular mechanism causing the MSI phenotype in sporadic CRC is the biallelic inactivation of *MLH1* by hypermethylation of the promoter [39]. CRC with hypermethylation of *MLH1* are frequently accompanied by the CpG island methylator phenotype. *MLH1*-hypermethylated CRC are, in line with an MMR defect, highly enriched for frameshift mutations in long mononucleotide repeats and hypermutated [40]. For a subset of the sporadic MSI CRC, two acquired somatic events explain the loss of MMR activity. In these cases, two somatic hits affecting both alleles of *MLH1* or *MSH2* are identified, which have caused the MSI phenotype in the CRC [41–43]. In MSI CRC derived from both LS as well as sporadic cases, the somatic inactivation of the MMR machinery enables the accumulation of insertions and deletions in repetitive DNA sequences, which will eventually drive the development of MSI CRC.

## Frameshift mutations drive the development of MSI CRC

As discussed above, the inactivation of the DNA MMR pathway is established differently in sporadic and LS CRC. Next to the CpG island methylator phenotype in sporadic MSI CRC, another somatic difference between sporadic and familial MSI CRC has been established. The activating p.V600E hotspot mutation in *BRAF* is strongly associated with MMR-deficient CRC [44, 45], but is only observed in sporadic MSI CRC and not in MSI CRC derived from LS patients [46]. Nevertheless, the somatic alterations driving tumorigenesis after the inactivation of the DNA MMR pathway appear to be comparable in sporadic and familial MSI CRC. After the loss of MMR activity, somatic misincorporations, insertions and deletions are rapidly accumulated. It has been established that, on average, approximately 1300 somatic base substitutions are acquired in MSI CRC derived from LS patients, whereas only 190 somatic base substitutions are present in microsatellite stable (MSS) tumors [47]. Similarly, sporadic MSI CRC has a significantly increased number of base substitutions compared to MSS CRC. In addition to these base substitutions, large numbers of somatic insertions and deletions are observed in MSI CRC. A subset of these insertions and deletions can affect coding regions of the genome, potentially resulting in frameshifts in the open reading frame of genes. These frameshift mutations can also occur in specific tumor suppressor genes which are susceptible for these mutations since they harbor repetitive DNA sequences in their coding regions [48]. For example, frameshift mutations are frequently observed in the mononucleotide repeats of tumor suppressor genes *APC* [49], *BAX* [50], and *TGFBR2 [* 51] in MSI CRC. These somatic frameshifts in *APC*, *BAX* and *TGFBR2* are observed in approximately 70, 50 and 90 % of the MSI CRC, and loss of the functional expression of the encoded tumor suppressor proteins can drive tumorigenesis [49–51].

In recent years it has been appreciated that frameshift mutations vary between patients and tumors. Within each tumor new self-antigens, the so-called neoantigens, accumulate. Indeed, an accumulation of approximately 40 unique epitopes for all MHC class I molecules per individual colorectal cancer has been observed [52]. They arise as a by-product when frameshifted neoproteins are degraded and derived peptides are presented. Neoantigens are therefore individual, immunogenic peptides, and were shown to contribute to a better survival of patients while being exploited as targets for immunotherapy (as reviewed in [53]). Frameshift mutations, however, do not always lead to neoantigen production. Nonsense-mediated mRNA decay, the surveillance pathway to reduce errors in gene expression by mRNA transcripts, eliminates aberrant mRNAs that encode incomplete polypeptides [54].

## Immunological responses against MSI CRC

High numbers of TILs represent a common hallmark of CRC and especially MSI tumors [55, 56]. Extensive in vitro and in vivo studies involving histopathological, phenotypical and molecular characterization confirmed that especially T helper cells and CTLs are attracted to the tumor tissue and are reactive against specific tumor epitopes [56–62]. Intriguingly, these T cells are specific for neoepitopes arising from frameshift mutations. Next to the previously mentioned tumor suppressor genes *APC* (involved in the Wnt pathway)*, BAX* (apoptosis-related) and *TGFBR2* (involved in signal transduction), frameshift mutations are also frequently identified in the genes *TP53* (plays a role in apoptosis, genomic stability, and inhibition of angiogenesis), *OGT* (involved in protein translocation and modification) and *CASP5* (role in inflammation) [34, 49–51, 58–61, 63–65]. Specific CTL responses have been observed for neopeptides derived from mutant OGT [64], MSH3 (−1) [66], TGFβRII [60, 67] and caspase-5 [58] proteins. Importantly, it has been shown that the density of TILs positively correlates with the amount of frameshift neoantigens presented by the tumor [68, 69].

Compared to MSS, tumoral DC in MSI was shown to express increased levels of co-stimulatory molecules, which are necessary for a proper T cell activation. This is probably caused by the high immunogenicity of MSI tumors, which express increased levels of immune-activating molecules such as heat shock proteins and proinflammatory cytokines making them extremely efficient in triggering DC activation [70].

Furthermore, upregulation of the integrin molecule CD103 on CD8+ T cells, which is only found in MSI tumors, equips CD8+ T cells with a highly tumor infiltrative capacity not observed in MSS tumors [71]. In accordance to this, Belt et al. [72] indicated that in patients with stage II and III MSI CRC, a higher number of lymph nodes could be detected during resection. A high lymph node yield is an indicator for a lower disease recurrence rate and a better disease-free survival and is suggested to be correlated to lymphocyte infiltration and potent anti-tumor responses. Elevated concentration of granzyme B and perforin in tumor-infiltrating CD8+ T cells underline their reactive status in MSI tumors [55, 70, 73].

In addition, specific CD4+ T cell responses have been observed in patients with MSI CRC and TGFβRII-specific CD4+ T cells could be expanded from MSI tumors and patient blood. At the same time, dense CD4+ T cell infiltration was observed in MSI tumors [61]. In some patients, CD4+ rather than CD8+ T cells, even dominated the tumor-infiltrating T cell response. In these patients, IFN-y responses against several MSI antigens were observed, although it was not specified whether these responses were derived from CD4+ or CD8+ T cells [57]. Another evidence for the importance of tumor-infiltrating CD4+ T cells in MSI CCR comes from a histological study. Here, the highest faction of CD4+ T cells was observed in tumors that downregulated MHC class II expression due to inactivating mutations [74]. This suggests that the infiltration of CD4+ T cells into the tumor leads to the preferential outgrow of MHC class II negative mutated cells. CD4+ T cells might contribute significantly to tumor control by tumor antigen recognition via MHC class II on the tumor cells or indirectly by providing T cell help to CD8+ T cells after being activated by antigen-presenting cells presenting released tumor antigens in MHC class II. Indeed, CD4 T cells in MSI tumors have been found to secret high amounts of pro-inflammatory cytokines which positively influenced the anti-tumor response [70]. This concerted action of both T helper cells and CTL is seen as prerequisite for mounting an effective anti-tumor immune response and correlated with a higher survival of MSI CRC patients [61, 75].

Regulatory T cells (Tregs) may negatively affect the anti-tumor response by suppression of CTL. The absence of Tregs in the tumor is therefore desired in order to stimulate tumor regression. It has been observed that Tregs increase CD103 expression in MSI tumors and are able to infiltrate MSI, but not MSS tumors in high amounts. This Treg infiltration is negatively correlated with an efficient anti-tumor CTL response [76, 77] which was also shown by the finding that a higher ratio of CD8+ T cells to Tregs correlates with better outcome in MSI CRC [78]. The ratio of Tregs to CD8+ T cells could therefore be used as prognostic marker in patients with MSI tumors. Following this line of thought, in vitro studies evaluating the impact of Tregs on CTL, specific for frameshift peptides, have shown that depletion of Tregs could promote CTL activity [79] and that antigens which are recognized by Tregs could have an impact on their ability to suppress CD8+ T cells. Contrary to these previous histology-based studies, La Gouvello et al. [80] revealed that FoxP3 mRNA expression levels, indicative for the presence of Tregs, are increased in MSS tumors compared with MSI tumors. Nevertheless, in the same study MSS tumors also showed increased levels of IL-6 and IL-17 when compared to healthy tissue and MSI tumors. In the context of IL-6, there is considerable plasticity between Tregs and IL-17 producing T helper cells (Th17 cells) possibly suggesting that the increased expression of FoxP3 in MSS tumors might have been caused by the presence of Th17 cells still expressing Foxp3, and not by actual Tregs [81]. These contrasting results indicate that more knowledge has to be gained to fully understand the role of Tregs in MSI and MSS tumors and that thorough analysis is only possible with multiparameter analysis of infiltrating immune cells.

**Fig. 1:**
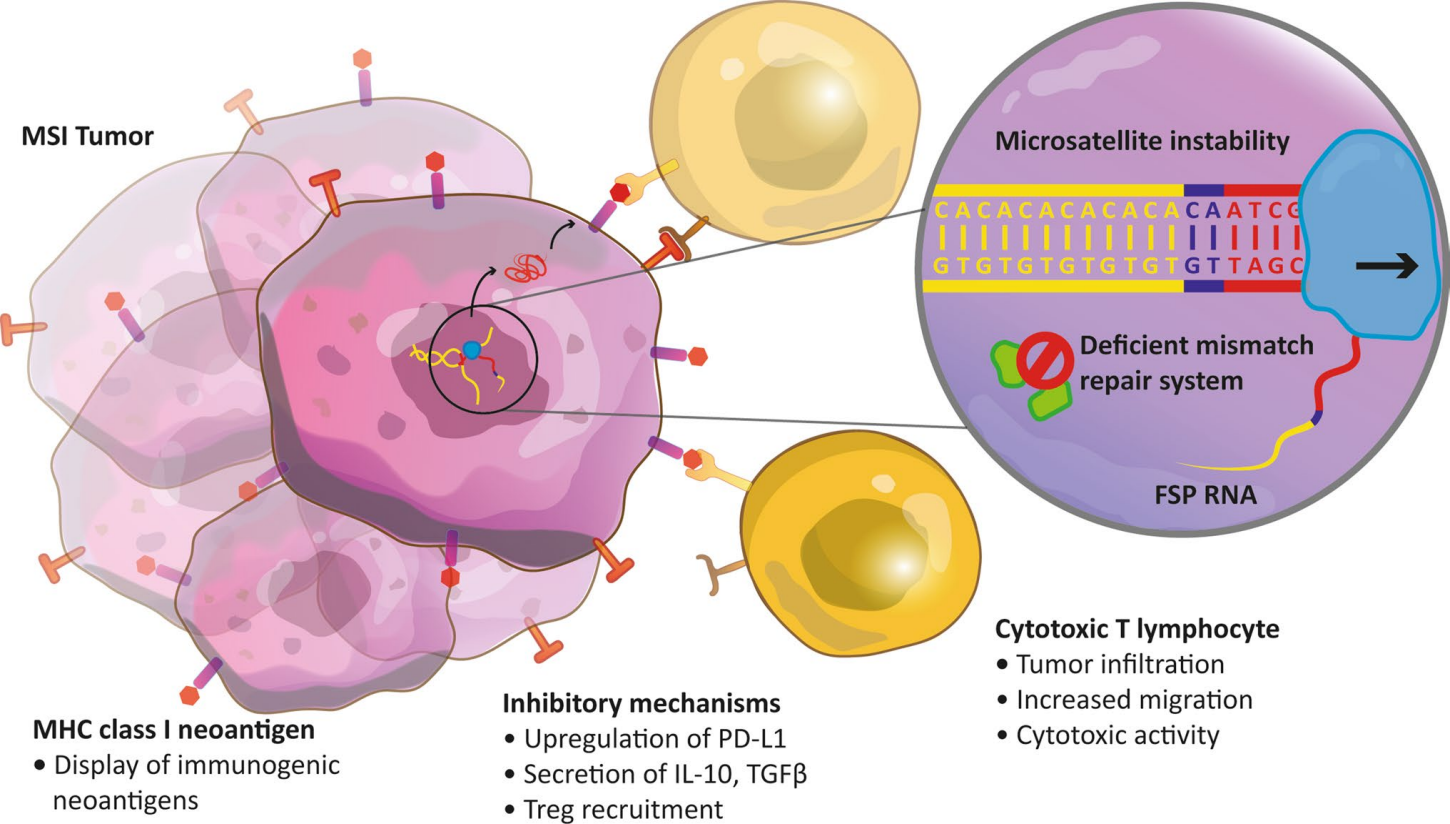
Molecular and immunological features of an microsatellite instable tumor harboring a frameshift mutation. A somatic insertion of a CA dinucleotide (*purple*) in a (CA)_6_ repetitive DNA sequence (*yellow*) has not been recognized and corrected due to mismatch repair deficiency. The CA insertion affects a protein coding exon and, therefore, the open reading frame of the encoded messenger RNA is altered (out-of-frame, *colored red*). Consequently, this results in the translation of a frameshift protein with a c-terminally altered amino acid sequence (not shown). *FSP* frameshift-derived peptide, *MSI* microsatellite instability, *Treg* regulatory T cell

Taken together, a successful anti-tumor immune response can be initiated if the tumor contains mutated proteins that are presented on MHC class I and recognized as nonself by T cells (Fig. 1). In turn, this activates the adaptive immune cells to combat the tumor via various strategies. Several immune cell types which have a positive effect on anti-tumor response, such as CD8+ T cells, are infiltrating MSI tumors but not MSS tumors. It is suggested that the highly immunogenic neoantigens of MSI tumors are causative for increased immune activity. As a consequence of this enhanced anti-tumor reactivity, patients with MSI tumors show a better prognosis and a higher survival. Nevertheless, the direct cause-and-effect relationship between frameshift neoantigens and functional neoantigen-specific TILs remains to be proven. Cell types important for inhibiting the immune response and promoting tumor growth, for example Tregs, are possibly increased in MSI tumors in contrast to MSS tumors. Further elucidation of Treg, but also macrophage, NK cell, B cell, and myeloid-derived suppressor cell functions and their implications in MSI and MSS CRC is certainly necessary.

## Therapy outlook in MSI CRC

Current treatment of CRC is mainly based on surgery, radiotherapy and/or chemotherapy, depending on the stage of disease. Inducing durable responses to these treatments remains challenging due to differences in patient characteristics, like age and comorbidity, but also unknown genetic influences and differences in tumor immune evasion. Interestingly, the group of Lynch showed already in 1997 that patients with LS compared to patients with similarly staged sporadic CRC have a significantly higher chance of survival in a period of 5 years [82]. Due to the strong neoantigen-based immune responses found in germline and MSI CRC patients, immunotherapy aiming at extending and strengthening these responses holds great potential.

Recently, two studies analyzed the effects of MSI tumor microenvironment and inhibitory molecules on neoantigen-specific TILs [83, 84]. The group of Housseau reported that MSI tumors are not totally eliminated despite the high amounts of infiltrating TILs. They showed that a high amount of immune inhibitory molecules (immune checkpoints) are expressed by MMR-deficient tumors and their microenvironment, notably PD-1, PD-L1, CTLA-4, lymphocyte-activation gene 3 and IDO. This was studied by performing immunohistochemistry, laser capture microdissection/qRT-PCR, flow cytometry and functional analysis. Consequently, they concluded that MSI tumors which express high amounts of neoantigens upregulate inhibitory molecules as a manner to counterbalance the high amount of infiltrating immune cells. Subsequently, they suspected that immune checkpoint inhibitors could be exclusively effective in this subtype of CRC [83]. Le et al. confirmed this theory in a phase I clinical trial. They noted that only one out of 33 CRC patients responded to PD-1 monoclonal antibodies [85, 86], which led them to further investigate the cause of this single response. It turned out that this patient had an MMR-deficient CRC. Subsequently, a phase II clinical trial was initiated to test the efficacy of PD-1 blockade with an anti-PD-1 monoclonal antibody on MSI and MSS CRC patients, which emphasized a clear benefit of treatment by PD-1 inhibition for patients with MSI CRC [84]. Other groups emphasized that MSI tumors also harbor other immune escape mechanisms to prevent effector T cell responses, like loss of MHC class I and II expression [87, 88]. Moreover, secretion of interleukin-10 and TGFβ at the tumor site was shown to act immunosuppressively (as reviewed in [89–91]). This indicates that inhibiting immune suppression by immune checkpoint inhibition could lead to improved treatment outcome in MSI CRC patients.

Another strategy to exploit the immune system for the fight against CRC is based on neoantigen vaccines and has received increasing attention in the last 5 years (as reviewed in [92, 93]). Neoantigens are not expressed in the thymus, and therefore lack negative selection, and are more specific than other tumor-associated antigens. In brief, neoantigen vaccines are developed by analyzing the genotype of tumor cells and predicting highly immunogenic tumor neopeptides either by functional tests or by in silico prediction algorithms. Subsequently, these epitopes can be used for short and long peptide-based vaccines, DC vaccinations, adoptive autologous T cell transfer, and gene-modified cell therapies. In the latter two, ex vivo expanded anti-tumor neoepitope-specific T lymphoctytes or gene-modified T lymphocytes, respectively, could be administered to treat cancer patients.

Several groups have focussed on the optimization of neoantigen-epitope prediction algorithms in silico which make it possible to predict MHC class I, and to a lesser extent, MHC class II tumor neoepitopes and their interaction strength with the T cell receptor [94]. However, the prediction algorithms for the affinity of the T cell receptor-MHC class II complex are not fully validated yet. By exploiting these tools, it has been described that neoantigen vaccination can be used to reduce tumor growth in vivo. For instance, the genetic makeup of the B16F10 melanoma mouse model predicted potential immunogenic neoepitopes. Vaccination with these neoantigens could increase tumor-specific T cell immunity in vivo in contrast to standard melanoma differentiation antigens, such as gp100, tyrosinase or TRP2 [95]. Moreover, in three murine tumor models a significant fraction of cancer mutations were shown to be immunogenic. Mostly, this unique set of mutations, the mutanome, was recognized by CD4+ T cells and a vaccination approach with antigens recognized by CD4+ T cells indeed resulted in strong anti-tumor activity [96]. CD4+ T cells might directly affect tumor growth via MHC class II or in MHC class II negative tumors CD4+ T cells might provide T cell help to CTL. Similar results were obtained when studying neoantigen vaccination in the setting of cancer immunoediting in mice sarcomas in combination with checkpoint inhibition. This project revealed that highly immunogenic neoantigens are essential for the anti-tumor immune response after checkpoint inhibition and can promote cancer immunoediting [97, 98]. Furthermore, the presence of certain neoepitopes within the neoantigen landscape in malignant melanoma patients was important for CTLA-4 blockade-mediated anti-tumor immunity and could be used as a predictive marker for ipilimumab treatment [99]. Finally, a recent publication describes the success of inducing neoantigen-specific patient T cells against melanoma by DC vaccination after vaccine candidate prediction and immune response monitoring [100].

Due to high frequencies of non-synonymous mutations, the presence of frameshift-mutated neoproteins, and strong effector T cell infiltration with tumor eradication, MSI CRC emerged as an important model system for (neoantigen-based) immunotherapy in therapeutic and protective settings. We initially focussed on a clinical study with DC loaded with the tumor-associated antigen carcinoembryonic antigen (CEA). Sporadic metastatic CRC patients were vaccinated with CEA mRNA electroporated or CEA-peptide-pulsed DC. A benefit for CEA mRNA electroporation over peptide-pulsing was not observed [101, 102]; however, in the majority of patients vaccinated with peptide-pulsed DC, CEA-specific T cell responses could be demonstrated. The low number of patients in these studies did not allow any correlation between immunological results and clinical outcome [101, 102]. Building on these results, we set up a clinical trial focusing on LS patients consisting of two groups of subjects: individuals of group I) carry a germline MMR gene mutation and had an MSI CRC. Group II) individuals carry a germline MMR gene mutation without manifestation of CRC. The aim of this study (NCT01885702) is to evaluate whether peptide-loaded DC can induce or enhance an immune response to CEA (YLSGANLNL), as well as, two frameshift-derived neoantigens of TGFβRII (RLSSCVPVA) and caspase-5 (FLIIWQNTM). Neoantigen-based vaccinations are studied in another clinical trial (NCT01461148) recruiting patients with surgically resected MSI CRC with lymph node metastases or metastasis to one or more distant organs.

**Fig. 2:**
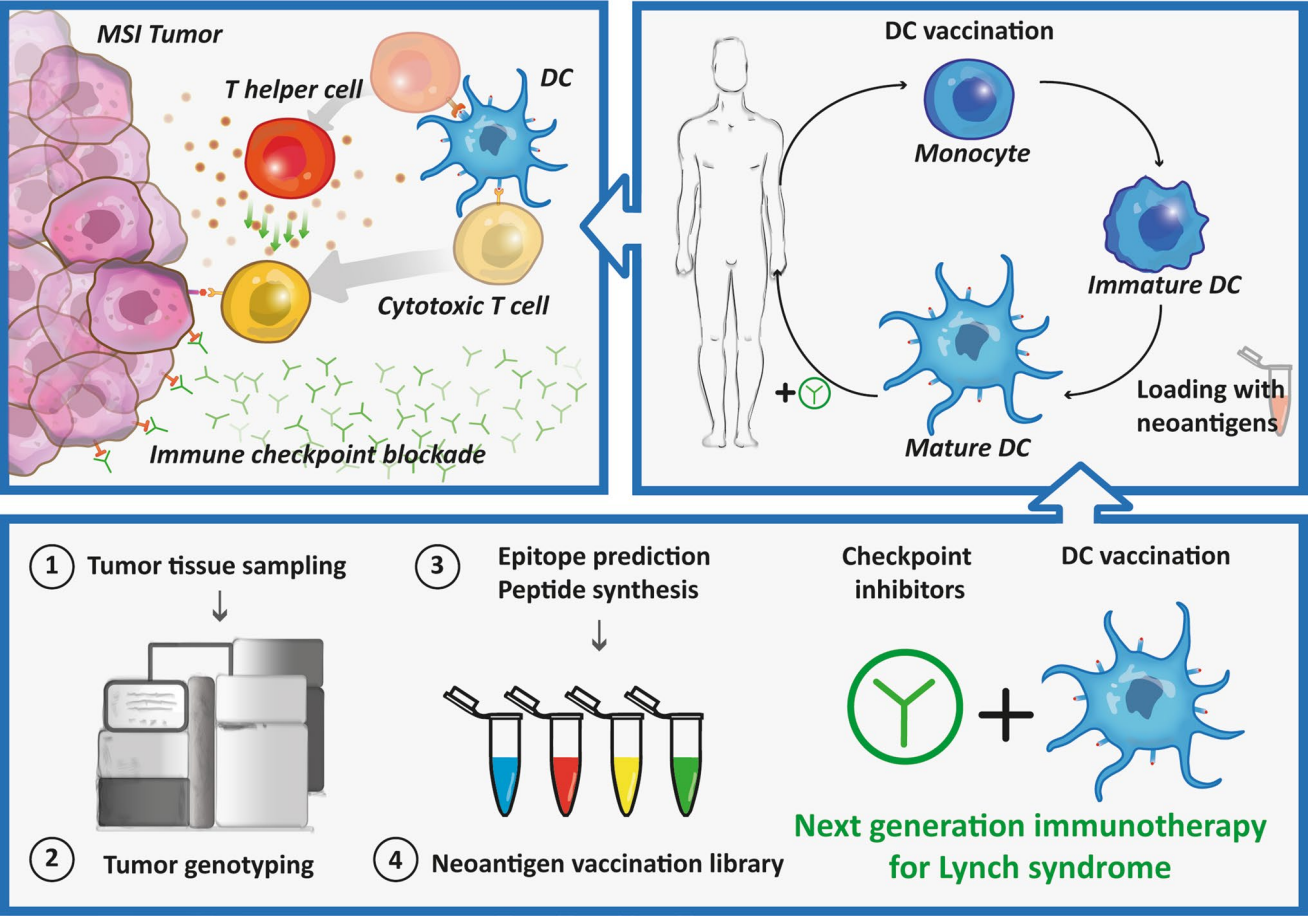
Therapeutic approach for Lynch syndrome mutation carriers combining immune checkpoint inhibitors and DC vaccination*. MSI* microsatellite instability

While these clinical trials indicate neoantigen vaccination as an individualized therapy with high future potential, several considerations have to be made regarding its optimization. Firstly, it should be investigated if induced neoantigen-specific T cell responses are clinically relevant for the cancer patient, resulting in a long-lasting tumor control. Particularly, in highly immunogenic tumors as melanoma, lung cancer or CRC the mutation turnover might be enhanced and could lead to changes of neoantigens expression in time, hampering clinical efficacy vaccination against predefined neoantigens. This might be overcome by preventive treatment or treatment at an early stage before novel mutations arise and before possible loss of MHC molecules. Moreover, multiple neoantigens could be targeted to prevent antigen loss, to avoid tumor escape and to combat tumor heterogeneity. Secondly, the optimal delivery method of the vaccine should be explored. With the rise of nanoparticle-mediated delivery systems or advanced antibody engineering, several methods exist to target specific cells of the immune system and prevent side effects [103]. Finally, it has to be investigated how cost-effective and resource demanding neoantigen-based vaccination is. Particularly, for patient-specific vaccination the treatment schedule, spanning a timeframe from cancer diagnostics to the production of the final personalized vaccine, has to be taken into account. In order to overcome current limitations of treatment for patients with MSI tumors, it could be beneficial to combine neoantigen-based immunotherapy with immune checkpoint inhibition (Fig. 2). This would not only provide selection and activation of neoantigen-specific T cells, but would also remove tumor-mediated immunosuppression.

Overall, this Focussed Research Review describes that patients with MSI CRC show highly increased mutation rates and expression of immunogenic frameshift neopeptides due to an inefficient MMR system. In turn, this positively coincides with extensive infiltration of the tumor by activated neoantigen-specific cytotoxic and T helper cells, resulting in an anti-tumor immune response and enhanced patient survival in contrast to patients with MMR-proficient MSS CRC. It is clear that one has to remove the barriers for CTL induced by DC vaccines, to reach the tumor and properly exploit their effector functions. For that to occur, immunosuppressive networks must be eradicated. An approach to address these issues is the combination of DC vaccine candidates and immune checkpoint inhibitors, which can abolish the means by which the tumor tries to dampen the immune response. Ultimately, antigen-specific vaccination strategies are expected to remain important next to less specific checkpoint inhibitors, to obtain curative immunotherapies.

## Abbreviations

CEA: Carcinoembryonic antigen
CRC: Colorectal cancer(s)
LS: Lynch syndrome
MMR: Mismatch repair
MSI: Microsatellite instability
MSS: Microsatellite stable
Treg: Regulatory T cell
